# Estimating miners at risk for occupational noise-induced hearing loss: A review of data from a South African platinum mine

**DOI:** 10.4102/sajcd.v67i2.677

**Published:** 2020-03-26

**Authors:** Liepollo Ntlhakana, Gill Nelson, Katijah Khoza-Shangase

**Affiliations:** 1 Department of Speech Pathology and Audiology, Faculty of Humanities, School of Human and Community Development, University of the Witwatersrand, Johannesburg, South Africa; 2 Occupational Health Division, School of Public Health, Faculty of Health Sciences, University of the Witwatersrand, Johannesburg, South Africa; 3 Institute for Global Health, University College London, London, The United Kingdom

**Keywords:** Occupational exposures, Risk factors, Electronic data, Hearing conservation programme, Hearing loss, Miners

## Abstract

**Background:**

Occupational noise-induced hearing loss (ONIHL) is a complex, but preventable, health problem for South African miners. Meticulously collected data should be made use of to design interventions to address this health issue.

**Objectives:**

A single mine’s electronic data were reviewed in a secondary data review to determine, from the records, factors that hearing conservation practitioners deemed useful for identifying ‘at risk’ miners and to establish factors that would pave the way for the integration of the 2014 hearing conservation programme (HCP) milestones into the mine’s current proactive data management system (PDMS). The objectives of this article were to establish how miners with published risk factors associated with ONIHL were managed by the mine’s hearing conservation practitioners as part of the HCP; to determine if the mine’s hearing conservation practitioners could estimate miners’ risk of ONIHL using baseline percentage loss of hearing (PLH) as a hearing conservation measure; and to estimate the contribution of noise exposure to ONIHL risk.

**Method:**

In a secondary data review design, records in a platinum mine’s two electronic data sets were reviewed: the first contained diagnostic audiometry records (*N* = 1938) and the second comprised a subset of miners diagnosed with ONIHL (*n* = 73). Data were available for the period 2014–2017 and included demographic, occupational, audiometry and ONIHL diagnosis data. Miners’ risk factors associated with ONIHL were identified using the functional risk management structure. A logistic regression model was used for the baseline PLH margins of 0% – 40% (in 5% increments) to estimate the adjusted predictions for miners at risk of developing ONIHL. The contribution of noise exposure as a risk for ONIHL was estimated using a two-way sample proportion test.

**Results:**

The mean age of the miners (all male candidates) was 47 ± 8.5 years; more than 80% had worked for longer than 10 years. Valid baseline audiometry records were available for only 34% (*n* = 669) of the miners. Miners with a 0% baseline PLH had a 20% predicted risk of ONIHL, and a 45% predicted risk if they had a 40% baseline PLH – these employees were referred. The noise exposure risk rankings revealed that 64.9% (*n* = 1250) of the miners were exposed to 91 dBA – 105 dBA noise exposure levels and that 59 (80.8%) diagnosed with ONIHL were exposed to noise levels of up to 104 dBA.

**Conclusion:**

These findings indicate significant gaps in the mine’s PDMS, requiring attention. Nonetheless, the mine’s current data capturing may be used to identify miners at risk of developing ONIHL. The PLH referral cut-off point (≥2.5%) used by the mine’s hearing conservation practitioners, when used in conjunction with baseline PLH shifts, was the major factor in early identification of ONIHL in miners exposed to ≥85 dBA noise. An inclusive integrative data management programme that includes the medical surveillance data set of the miners’ noise exposure levels, occupations, ages and medical treatments for tuberculosis and human immunodeficiency syndrome is recommended, as these are important risk indicators for developing ONIHL, particularly within the South African context.

## Introduction

The World Health Organization (WHO) reported a 16% prevalence of occupational noise-induced hearing loss (ONIHL) in 2010 (WHO, 2010). In South Africa, ONIHL is the third most commonly reported occupational disease affecting miners (Balfour-Kaipa, 2014). Individuals with ONIHL are susceptible to sleep disturbances, psychological stress associated with the frustration of losing hearing function, fatigue and cardiovascular problems (Malatji & Stewart, 2013). Workers’ susceptibility to ONIHL is exacerbated by ageing, sex (Khoza-Shangase, 2019; Strauss, Swanepoel, Becker, Eloff, & Hall, 2012), a history of otalgia and middle ear infections (Edwards, 2008), previous diagnosis of sensory neural hearing loss, cigarette smoking, hypertension and type 2 diabetes (Hong, Lee, Park, & Kim, 2014). The risk of developing ONIHL is increased for workers currently on treatment for tuberculosis (TB), human immunodeficiency syndrome (HIV) and/or cancer (Khoza-Shangase, 2019). The increased risk of ONIHL is because of the synergistic effect of high noise levels and the other ototoxic factors mentioned above (Campo, Morata, & Hong, 2013; Hong et al., 2014; Khoza-Shangase, 2019). The symptoms of ONIHL include a sense of fullness in both ears, tinnitus, balance problems, difficulty following conversations and hearing warning signals, and progressive hearing loss in one or both ears (Feuerstein & Chasin, 2009).

In the diagnosis and management of ONIHL, it is important to include tracking and monitoring all the aforementioned risk factors to ensure efficient implementation of any hearing conservation programme (HCP). Occupational diseases in the mining industry, including pulmonary tuberculosis and silicosis, for example, have been well documented (Hermanus, 2007; WHO, 2010); however, little evidence exists on how these diseases are treated within HCPs, although synergistic effects of these diseases and their treatments with noise exposure on the auditory system have been raised (Khoza-Shangase, 2019). The authors of this article concur with Hermanus (2007) in arguing that such evidence is important, as it can assist in developing disease control policies for the mines and health service planning, including HCP planning, implementation and monitoring.

The aforementioned comorbid health conditions have not been considered in HCPs (Nelson, Nelson, Concha-Barrientos, & Fingerhut, 2005). In countries like South Africa, where the prevalence of ONIHL is high, it can be argued that the failure to meet ONIHL elimination targets is influenced by the lack of contextual responsiveness to the burden of disease. Consequently, HCPs have been deemed ineffective in preventing ONIHL in South Africa (Edwards & Kritzinger, 2012; Moroe et al., 2018; Ntlhakana, Kanji, & Khoza-Shangase, 2015), in spite of being introduced in South African large-scale mines as early as 1988 (Franz & Phillips, 2001).

A number of studies on HCPs in South African mines have reported factors associated with the failure of HCPs to prevent ONIHL. These include excessive noise levels emitted by equipment (higher than the legislated level of 85 dBA), unsatisfactory uptake on the use of hearing protection devices (HPDs) by miners (Hansia & Dickinson, 2010; Ntlhakana et al., 2015) and evidence of high prevalence rates of ONIHL because of a combination of occupational and non-occupational factors (e.g. age, race and sex) (Strauss, Swanepoel, Becker, Eloff, & Hall, 2014). Weaknesses in HCPs, including poor risk management techniques and flawed audiometric database analysis (ADBA) (Franz & Phillips, 2001), have been proposed as some of the priorities requiring attention by the mining authorities and the audiology community in South Africa (Moroe & Khoza-Shangase, 2018).

**FIGURE 1:**
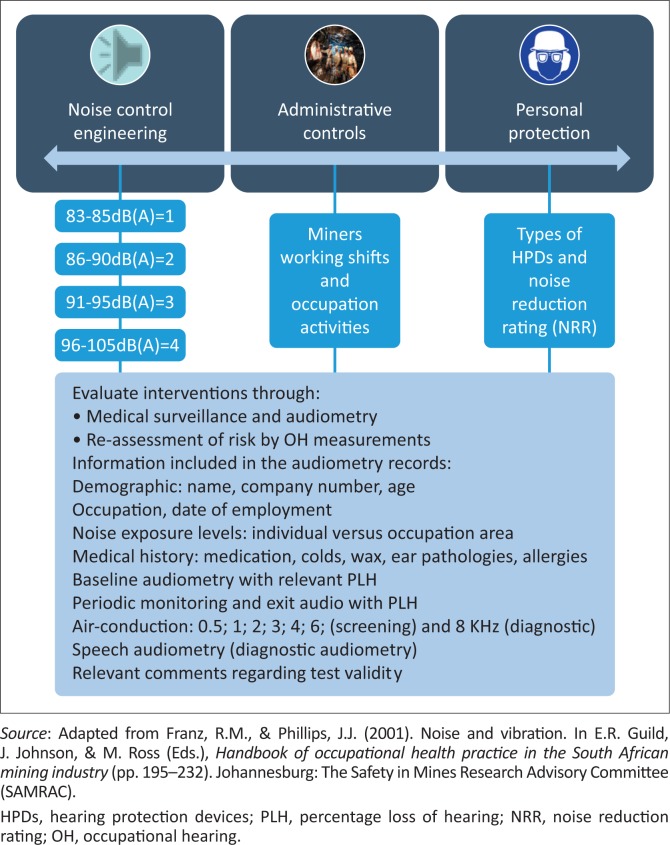
Functional structure of a risk management system for occupational noise.

The ADBA procedures were developed by the American National Standards Institute Working Group (ANSI S12/WGS12) (ANSI, 1991), with the goal of assessing the effectiveness of HCPs. The ADBA procedures can be utilised to ensure the success of HCPs by identifying potential problem areas and challenges in the HCP prior to employees developing significant hearing loss (Adera, Gullickson, Helfer, Wang, & Gardner, 1995), hence highlighting the need for careful longitudinal tracking and monitoring of employees’ hearing function in relation to their noise exposure levels and other key risk factors. Such methods require comprehensive and consistent data collection to allow for an accurate and systematic assessment of the effectiveness of HCPs, which is a complex challenge with any well-meaning, data-reliant management system. The fact that there is more than one ADBA method that can be used introduces another challenge when it comes to comparative analysis of findings within and between organisations. The use of the ADBA for evaluating the effectiveness of HCPs, for example, was found lacking in Adera et al.’s (1995) paper, where ratings of HCPs were found to be inconsistent from year to year, and from procedure to procedure used. Use of such a procedure therefore is bound to present significant challenges, if all important and often contextually relevant factors (including the burden of disease specific to the region) do not form part of the data capturing and analysis processes. As illustrated in Figure 1, the technique includes recording the miners’ audiometry data from baseline to exit.

 This technique requires the use of a data management system to ‘identify occupations, workplaces and activities where hearing loss is progressing most, particularly in the case of a large workforce…..’ (Franz & Phillips, 2001, p. 209). Within the South African context, evidence suggests that although the ADBA is used, its effectiveness is questionable (Begley, 2006), possibly because of the factors listed above. Begley (2006) described how South African large-scale mines designed various versions of the ADBA, and how this inhibited the successful tracking of miners’ audiological status and the monitoring of those diagnosed with ONIHL. An HCP has seven pillars that include noise control engineering, noise measurement, administrative noise control measures, education–training–motivation, HPD usage, risk-based medical surveillance and audiometry. The technique described by Franz and Phillips focusses on three pillars, namely: (1) noise control engineering, (2) administrative noise control measures and (3) use of personal protection, as a proactive risk management tool for early identification and prevention of ONIHL; the said technique was reportedly adopted by the mine where the current study’s data were located. This technique is rather outdated, and there are more recent and more comprehensive HCP structures that cover all pillars of HCPs, such as complex interventions (Moroe, 2018); nonetheless, this was the technique that was followed for the secondary data analysed in the current study. This method reportedly allowed the mine to send ONIHL cases to their insurance company according to Instruction 171.

We analysed data from the mine’s medical surveillance and audiometry records to describe the mine’s ONIHL prevention efforts (Figure 1).

A proactive data management system (PDMS), as a single point of contact for organisations, has been used for many years to understand complex organisational trends. The PDMS follows best practice principles, allows for efficient monitoring of key factors identified by the company and provides intervention measures where required (Jantti & Cater-Steel, 2017). In South Africa, large-scale mines have attempted to use the ADBA technique that draws data from the mine’s medical surveillance and audiometry records, as a PDMS for the management of miners exposed to hazardous noise emitted by their equipment, to build the miners’ audiometry database and to prevent ONIHL. However, the ADBA’s failure is demonstrated by high noise levels and the continued reporting of miners with ONIHL (Balfour-Kaipa, 2014; Chamber of Mines, 2016). Thus, South African mines have not been successful at designing PDMSs to prevent ONIHL.

Previously reported high incidences of ONIHL in the South African mining industry indicate the miners’ hearing loss as a function of percentage loss of hearing (PLH) at 10% or greater since 2001 when the *Compensation for Occupational Injuries and Diseases Act, 1993 (Act No. 130 of 1993)* (COIDA) gazetted Circular Instruction 171 was instituted (Department of Labour, 2001). However, the fact that PLH calculations included only 500 hertz (Hz), 1000 Hz, 2000 Hz, 3000 Hz and 4000 Hz meant that higher frequencies known to be affected early by excessive noise exposure (as in ONIHL) were excluded. Hence, studies that reported on miners’ hearing function associated with ONIHL included low and high frequencies (500 Hz – 8000 Hz) (Khoza-Shangase, 2019; Moepeng, Soer, & Vinck, 2017; Strauss et al., 2012) in order to provide the miners a broader hearing function spectrum. However, for ONIHL compensation claim processing, which is still practised by the Compensation Commissioner, the miners’ audiometry records had to reflect the PLH score, indicating the miners’ hearing loss in spite of the noted weaknesses of using such scores.

This led to the review of HCP milestones by the Minerals Council South Africa, focussing on the mining equipment noise emissions and the tracking of the miners’ hearing thresholds (high-frequency standard thresholds) (Inspectorate, 2017). The new milestones set out in 2014 by the mining industry stakeholders stated that no individual miner’s standard hearing threshold shift (STS) should exceed 25 dB from baseline, in both ears, and that the total operational or process noise emitted by any equipment must not exceed 107 dBA, by the year 2024 (Mine Health and Safety Council – MHSC, 2015). The use of the STS as a sensitive measure for early detection of hearing loss was deemed a proactive practice of managing miners at risk for ONIHL. This initiative prompted the mines to integrate the miners’ newly recorded standard thresholds and threshold shifts (2014–2016) into their current reporting systems in order to improve their efficiency in identifying miners ‘at risk’ of developing ONIHL and to be in line with WHO targets for ONIHL (WHO, 2010). We reviewed the data recorded by the mine in order to determine, from the records, factors that hearing conservation practitioners deemed useful for identifying ‘at risk’ miners and to establish factors that would pave the way for the integration of the 2014 HCP milestones into the current PDMS of the mine.

The objectives of this article were to establish how miners with published risk factors associated with ONIHL were managed by the mine’s hearing conservation practitioners as part of the HCP; to determine if the mine’s hearing conservation practitioners could estimate miners’ risk of ONIHL using baseline PLH as a hearing conservation measure; and to estimate the contribution of noise exposure to ONIHL risk.

## Methods

### Research design

This was a secondary data review of miners’ electronic records from a platinum mine in Limpopo province, South Africa. Secondary data are called as such for various reasons, including the fact that the data were obtained by somebody else; the data had already undergone one layer of analysis prior to the secondary analysis; and that the data were collected for a focus or objective different to the one these are currently used for (Sorensen, Sabroe, & Olsen, 1996). This is an identified limitation of the current design; however, the limitation was in itself a key aspect of the study’s objective of assessing how the mine utilises its data management system as it is for its HCP.

### Study population

We reviewed all miners’ electronic medical surveillance and audiometry records at the research site. This comprised records including miners’ age, sex, years of experience, occupations, noise exposure levels, medical surveillance and audiometric test results.

### Data collection and data management

The records reviewed in this secondary data review were stored in two databases, and comprised data from 2014 to 2017. The main database contained 1963 medical surveillance and diagnostic audiometry records of miners, all of which had a baseline PLH shift of ≥2.5% (value set by the mine to refer miners for diagnostic audiometry), and had been referred to an occupational medical practitioner for further intervention, and to an audiologist for diagnostic audiometry. The second data set was a subset of records of 73 miners who had calculated PLH shifts of ≥10% from baseline. Following the diagnostic audiometry assessment, a diagnosis of ONIHL was confirmed by the audiologist, and some miners had been presented to the Rand Mutual Assurance company for ONIHL compensation. These two sets of data were handled separately.

All data underwent data cleaning processes that included the removal of duplicate records (*n* = 25), after which 1938 unique records remained in the main database. The audiometric records contained demographic, occupational and audiometry data (screening and diagnostic), and ONIHL diagnosis information. Careful scrutiny and recording of the process followed for miners in both data sets in relation to risk factors was performed for analysis.

It should be noted that because this is a secondary data review, PLH values were what was recorded and therefore reviewed, but it is acknowledged that the South African mines had started moving towards the new HCP milestones (2014) of using STS.

### Data analysis

Extracted data were captured into Microsoft Excel, after which they were imported into and analysed using Stata (version 15.1). The variables, such as age, baseline PLH, diagnostic audiometry PLH, years of noise exposure, noise exposure levels and recorded risk factors, were analysed using descriptive statistical analysis, logistic regression analysis, and a two-way sample proportions test (z-test).

To establish how the mine’s hearing conservation practitioners managed miners presenting with risk factors associated with ONIHL, risk factors stated in the functional risk management structure (Figure 1) (Franz & Phillips, 2001) were used.

To determine if the mine’s hearing conservation practitioners could estimate miners’ risk of ONIHL at baseline, PLH margins from 0% to 40% (5% increments) were used to estimate and interpret adjusted predictions, using logistic regression analysis. Finally, the contribution of noise exposure to the risk for ONIHL was estimated using a two-way sample proportion test (*z*-test), with the significance level set at 95%.

### Ethical considerations

Ethical clearance to conduct the study was obtained from the University of the Witwatersrand’s Human Ethics Committee (M180273) on 11 April 2018, and permission to access the records was obtained from the management team of the mining company. The study adhered to the Declaration of Helsinki 1975, as revised in 2008, as far as ethical considerations were concerned.

## Results

### To establish how miners with risk factors associated with occupational noise-induced hearing loss were managed within a hearing conservation programme

The workforce comprised only male mine workers. Tables 1 and 2 illustrate the miners’ ages and years of working in the mine, with a mean age of 47 (± 8.5) years (44–55 years) and a working experience of 10 (± 3.5) years (8–13 years). No records of all risk factors, particularly medical conditions important for hearing monitoring such as TB and HIV (ototoxicity), were recorded in the audiology records. Audiology data captured in the mine’s ADBA system only included pure-tone audiometry.

**TABLE 1:** Demographic characteristics of the miners in the main subset (*N* = 1938).

Characteristic	*N*	%
Age (years)†		
20–30	40	2.1
31–40	273	14.1
41–50	594	30.6
51–60	1023	52.8
≥ 61	8	0.4
Working experience (years)‡		
1–5	162	0.8
6–10	1010	52.3
11–15	636	32.9
≥ 16	22	0.1

† , *n* = 1938;

‡ , *n* = 1930 (eight records with missing data).

**TABLE 2:** Demographic characteristics of the miners (*N* = 73).

Characteristic	*N*	%
Age (years)†		
20–30	0	0
31–40	11	15.1
41–50	38	52.1
51–60	24	33
≥ 61	0	0
Working experience (years)‡		
1–5	5	6.8
6–10	29	39.7
11–15	32	43.8
≥ 16	7	9.6

† , *n* = 73;

‡ , *n* = 73.

The data seem to indicate that hearing conservation factors included in the mine’s PDMS to manage noise exposure and miners at risk for ONIHL were PLH scores for screening and diagnostic audiometry, with categorised referral points for the miners, as ≤2.5%, 5%, 7% and ≥10%, to monitor those miners at risk for ONIHL and for compensation purposes.

Risk rankings for noise exposure levels were not available, from lowest to highest (1–4), for all the miners. Furthermore, only 34% of the miners (*n* = 669) had baseline audiometry records; periodic audiometry records were available for review but there were no monitoring audiometry records; and the diagnostic audiometry records included only pure-tone air-conduction results with no speech audiometry data.

### To determine if the mine’s hearing conservation practitioners could estimate miners’ risk of occupational noise-induced hearing loss at baseline

**FIGURE 2:**
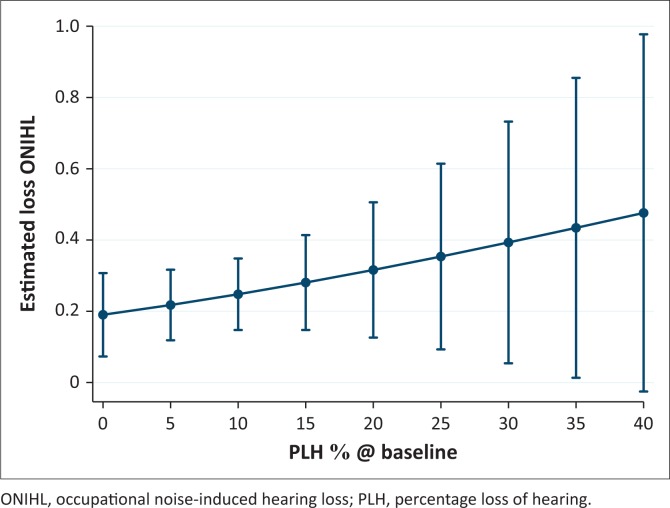
Baseline percentage loss of hearing adjusted predictions with 95% confidence intervals for occupational noise-induced hearing loss.

The miners’ estimated risk for ONIHL was based on the baseline PLH shift of 2.5% used by the mine. Baseline PLH is core to tracking miner’s hearing deterioration and is required for the calculation of the PLH shift for compensation; to determine a PLH shift of >10%. Figure 2 illustrates baseline PLH margins that were generated through logistics regression, from 0% to 40%, used to distinguish (in increments of 5%) between miners at high and low risk of ONIHL. At 0% PLH at baseline, the risk of ONIHL was predicted to be 20%. A 20% PLH at baseline corresponded to a risk level for ONIHL of about 30%; and a risk of ONIHL was predicted to be around 45% when a miner had a baseline PLH of 40%. The baseline PLH shift of 2.5% used by the mine’s hearing conservation practitioners is a proactive measure used for the miners’ hearing preservation. Baseline PLH scores and PLH scores at ONIHL diagnosis included in the model for the second data subset (*N* = 73) were used to estimate the predicted risk for ONIHL.

### To estimate the contribution of noise exposure to occupational noise-induced hearing loss risk as a proactive measure

The miners’ noise exposure levels were recorded as continuous data and were aligned with their occupations. Table 3 shows the mine’s noise exposure risk rating categories that we formulated to classify miners at risk of ONIHL. More than 80% of the miners (*n* = 1578) were exposed to noise exposure levels that were higher than the legislated occupational exposure limit (OEL) of 85 dBA, with 64% of the miners falling into the two highest risk rating categories.

**TABLE 3:** Noise exposure risk rating categories of exposed miners (*N* = 1963).

Noise exposure level (dBA)	Noise exposure risk rating category	*N*	%
≤ 82	0 (low)	81	4.2
83–85	1 (medium)	279	14.4
86–90	2 (significant)	328	16.9
91–95	3	941	48.6
96–105	4	309	15.9
> 105	5 (high)	0	0

Fifty-nine (80.8%) of the 73 miners diagnosed with ONIHL were exposed to noise levels of 85 dBA – 104 dBA; 14 (19.2%) were exposed to noise levels of <85 dBA. Although both groups (those exposed to <85 dBA and those exposed to ≥85 dBA) were exposed to high noise levels, the results indicated that the risk for ONIHL increased with the noise exposure category. The *z*-test showed a mean difference of 62% in noise exposure levels (*p* = 0.001) between miners exposed to ≥85 dBA and those exposed to <85 dBA.

## Discussion

Our study was guided by Franz and Phillips’ (2001) risk management tool, which was introduced in the early 2000s. Analysis of the miners’ records revealed that the mine was using a siloed approach for risk assessment of noise hazards and identification of miners at risk of ONIHL. The miners’ baseline PLH scores could estimate miners’ risk for ONIHL, although the 2.5% PLH used by the mine’s hearing conservation practitioners to refer miners for further intervention delayed the process of identifying miners at risk of ONIH and that the OEL for noise, of ≥85 dBA, is not low enough to prevent ONIHL.

With regard to establishing how the mine’s hearing conservation practitioners proactively managed miners presenting with risk factors associated with ONIHL findings showed that miners older than 40 years and those who had worked in mining for longer than 6 years were at higher risk of developing ONIHL. The fact that they were referred for diagnostic audiometry with a screening audiometry PLH score of ≥2.5% meant they were suspected of being at risk of developing ONIHL. In other studies, conducted in South African mines (Khoza-Shangase, 2019; Strauss et al., 2012), age was identified as a risk factor for ONIHL, and our findings support this. Miners’ increasing age is already considered by the mines’ hearing conservation practitioners as a risk factor for ONIHL; increasing work duration could be similarly considered as a risk factor.

The ADBA technique used by the mines requires more sensitive measures to identify miners at risk of ONIHL. The South African mines consider annual and diagnostic audiometry procedures as sensitive measures for identification of hearing loss; hence, both audiometry procedures were conducted on miners working in areas identified as high risk for ONIHL. Although Franz and Phillips (2001) recommended the use of the ADBA technique, based on the findings from Royster and Royster’s (1986) study, the large-scale mines designed mine-specific ADBA techniques, guided by the Noise Induced Hearing Loss Regulation 171 (2001) and the SANS 10083:2013. According to both the regulation and SANS 10083:2013, two diagnostic audiogram results should be used to confirm the ONIHL diagnosis. Our review of the mine’s electronic records revealed that none of the miners had two diagnostic audiometry records, deeming these results incomplete and inconclusive for the diagnosis of ONIHL (Franz & Phillips, 2001). Although duplicate diagnostic audiometry records were not in the mine’s reviewed records, they could have been available and kept with other miners’ records. This undermined the ADBA effort to provide a full picture of the miners’ ONIHL diagnostic results.

In a review of gold miners’ hearing results, researchers argued for the importance of adding an objective diagnostic tool – the distortion-product otoacoustic emissions (DPOAEs) tool – for early detection of ONIHL (Moepeng et al., 2017). In addition, evidence from research on DPOAEs has provided insight into other causes that lead to occupational hearing loss, such as age, ototoxic drugs, chemical exposure, tuberculosis and HIV (Campo et al., 2013; Khoza-Shangase, 2019; Strauss et al., 2014). However, at the time of data collection for our study, the miners’ diagnostic audiometry data did not include information about TB and HIV treatment, as well as types of chemicals to which the miners were exposed at work. The 2014 HCP milestones recommended inclusion of the STS as a measure of the miners’ hearing function, with the aim of improving hearing loss identification for these populations, for which the mining industry needs to be commended.

The contextual implications for TB and HIV as contributory factors towards miners’ hearing loss cannot be ignored. Our review of the records showed that these data were not included in the miners’ hearing conservation data, in spite of several studies in the South African mining industry indicating that TB and HIV are associated with hearing loss (Hermanus, 2007; Khoza-Shangase, 2019). Previous research has argued for the inclusion of these conditions in the medical surveillance records of miners (Franz & Phillips, 2001; Khoza-Shangase, 2019; MHSC, 2015), as well as the audiometry records, for audiological monitoring. This should be performed as a proactive way of managing all risks associated with hearing loss.

In a recent record review of hearing function among gold miners, Khoza-Shangase (2019) found that miners with a history of TB treatment presented with worse high-frequency hearing loss than those without a history of TB treatment. The study also highlighted confounding factors that affect the auditory system in this population, namely, the use of ototoxic medications, age and noise exposure. Khoza-Shangase (2019) raised the importance of audiological monitoring for these miners to be integrated into the mines’ TB and HIV medical surveillance programmes in order to monitor hearing loss holistically. This recommendation was also made by Franz and Phillips (2001).

Our findings indicated that baseline audiometry referral scores used by the mines’ hearing conservation practitioners delay the early identification process and intervention of miners at risk of ONIHL. Even at 0% baseline PLH, the miners were at a 20% risk of developing ONIHL. In addition, the PLH scores provide low-frequency thresholds at baseline for the miners. The 2.5% PLH referral used by the mine was seen as proactive when compared to the legislated referral of 3.2% (SANS, 2013), and the method also provides hearing threshold information that could be used in conjunction with the STS to identify miners’ risk of developing ONIHL at baseline.

There is a dearth of literature on the use of baseline PLH as part of HCPs in South African industries where noise is a problem. The only other study that reviewed baseline PLH scores was conducted by Bronkorst and Schutte (2013) who reviewed audiometry records of workers in various occupational settings in the Western Cape province to highlight the importance of using the employees’ more sensitive baseline (B-baseline) audiometry results for hearing conservation purposes. The method recommended by Bronkorst and Schutte (2013) showed the value of baseline PLH and the potential use thereof without the need to recreate new baselines for the South African miners.

The miners who were exposed to excessive noise levels (≥85 dBA) had a greater chance of being diagnosed with ONIHL. Of the 73 miners diagnosed with ONIHL, 14 (19.1%) were exposed to noise levels <85 dBA. This implies that the OEL of ≥85 dBA used by the mines did not protect miners from developing ONIHL, or that there could be other risk factors that led to the miners’ ONIHL diagnosis. Our findings and those of Edwards et al. (2011) are in agreement as far as noise exposure levels in South African mines are concerned. However, we questioned the association of other risk factors with ONIHL, specific to miners exposed to <85 dBA noise levels. Thus, a recommendation is made for the hearing conservation practitioners in the mining industry to review the legislated OEL (≥85 dBA), and to consider comorbid factors associated with occupational hearing loss, in order to prevent ONIHL.

The fact that the participating mine did not record noise exposure risk rankings is problematic, and it limits efforts for the prevention of ONIHL. It is important for the mines to provide accurate noise exposure rankings in the miners’ audiometry records, specific to tasks and occupations, in order to correctly identify miners at risk of ONIHL. In addition, the noise measurement technologies and recording techniques used by the mines’ occupational health practitioners should be effective towards the prevention of ONIHL. Although the duration of exposure was not recorded in the current study, we acknowledge the importance of this aspect in ONIHL, over and above other personal factors such as genetic predisposition.

Although significant, the findings should be interpreted carefully, taking cognisance of methodological limitations. The data that we reviewed were exclusively drawn from one large-scale mine, from the miners’ diagnostic audiometry records, and revealed three critical limitations. Firstly, the shortcoming associated with the use of secondary data is that some interpretations of our findings are based on assumptions. Secondly, the occupational hygienist’s data were not included in the records that we reviewed; thus, additional exposures and duration thereof were not included in the data that we reviewed. Our results therefore reflect only a narrow view of the diagnostic audiometry data set. Thirdly, the miners’ medical surveillance records regarding TB and HIV status could not be accessed. This compromised our review, as we could not comment on the broader spectrum of the hearing health risks associated with the miners’ ONIHL. This exclusion of data from the medical surveillance database indicates that the mine did not necessarily view TB, HIV and their medications as risk factors for hearing loss and therefore did not consider them in their HCP strategies. Finally, the non-classification of noise exposure levels according to risk rankings (Franz & Phillips, 2001) implies that the mine may be applying a blanket intervention for miners exposed to noise levels of ≥85 dBA.

## Conclusion

Our findings, guided by the ADBA technique, which was recommended by Franz and Phillips in 2000, indicated that the mine was using an adaptation of a technique that was recommended more than 15 years ago. This resulted in the mine’s hearing conservation practitioners considering noise exposure as the sole occupational hazard for ONIHL. Other occupational exposures associated with ONIHL were excluded. Nonetheless, the effort towards keeping records for the miners at risk of developing ONIHL was commendable.

A lower baseline referral PLH score should be used to identify miners at risk of ONIHL early, also allowing for early intervention to be instituted. The mining company is encouraged to add the miner’s data on burden of diseases such as TB and HIV as part of the miners’ audiometry monitoring programmes. This would facilitate proactive management of miners at risk of developing any type of hearing loss, including ONIHL. The current data management system used by the mine has the potential to be integrated into, and used together with, the new 2014 HCP milestones, to monitor the miners’ hearing function across all frequencies. It is probable that the mines are not achieving the desired outcomes of tracking miners’ hearing loss accurately, because they are using the PLH levels to report miners at risk for ONIHL, rather than STS. We recommend that the mines implement the new milestones (use of STS) across all stages of HCP reporting, to ensure that all new baselines are based on STS levels. This will ensure early identification of ONIHL instead of focussing on PLH, which is concerned with compensation after a disability has occurred.
